# Model-Based Real-Time Non-Rigid Tracking

**DOI:** 10.3390/s17102342

**Published:** 2017-10-14

**Authors:** Sebastián Bronte, Luis M. Bergasa, Daniel Pizarro, Rafael Barea

**Affiliations:** Electronics Department, University of Alcalá, Campus Universitario, 28805 Alcalá de Henares, Spain; sebastian.bronte@depeca.uah.es (S.B.); daniel.pizarro@uah.es (D.P.); rafael.barea@uah.es (R.B.)

**Keywords:** SfT, tracking, PTAM, non-rigid reconstruction, SfM, NRSfM

## Abstract

This paper presents a sequential non-rigid reconstruction method that recovers the 3D shape and the camera pose of a deforming object from a video sequence and a previous shape model of the object. We take PTAM (Parallel Mapping and Tracking), a state-of-the-art sequential real-time SfM (Structure-from-Motion) engine, and we upgrade it to solve non-rigid reconstruction. Our method provides a good trade-off between processing time and reconstruction error without the need for specific processing hardware, such as GPUs. We improve the original PTAM matching by using descriptor-based features, as well as smoothness priors to better constrain the 3D error. This paper works with perspective projection and deals with outliers and missing data. We evaluate the tracking algorithm performance through different tests over several datasets of non-rigid deforming objects. Our method achieves state-of-the-art accuracy and can be used as a real-time method suitable for being embedded in portable devices.

## 1. Introduction

The problem of 3D reconstruction and camera localization from images is known as Structure-from-Motion (SfM). 3D awareness from visual cues is a natural task for a human being, but it is still a very challenging problem in computer vision. During the last few decades, SfM has been widely studied [1,2,3,4,5,6,7]. Thanks to modern computing capabilities (multicore CPUs, GPUs, etc.), current SfM algorithms are considered mature and capable of dealing with big volumes of data in real time [8,9].

The general assumption in SfM is based on the rigidity of the scene, where changes in the images are caused by the relative motion between the camera and the scene. Rigidity strongly links camera motion with image motion, making SfM a well-posed problem. Rigid SfM fails in scenarios where the rigidity assumption is violated. For instance, it fails to reconstruct scenes with multiple objects that move independently or with deformable objects, such as the human body, articulated objects, wires, flags, sheets, flesh, fabric, etc.

Reconstruction of deformable objects from images is known as Non-Rigid Structure from Motion (NRSfM). It has been actively studied in recent years [10,11,12,13,14,15]. When a prior model of the object is available, this problem is commonly known as Shape from Template (SfT) [16,17,18,19]. Current SfT/NRSfM methods lack the level of maturity of SfM.

In both SfM and NRSfM/SfT, we distinguish two categories of methods: batch approaches [10,11,12,13,20] and sequential approaches [21,22,23,24]. In the former, all data (images) are available beforehand and are jointly processed to obtain the 3D reconstruction. Those approaches are highly demanding in terms of processing time and memory, but achieve accurate reconstructions. In the second group, data are collected and processed online. This is usually a harder problem where less data are available for reconstruction than in batch approaches, resulting in less accurate reconstructions. However, sequential methods are the base for real-time implementations required in some interesting applications, such as Augmented Reality (AR) [25,26,27].

3D reconstruction from images is a key technology in many applications such as Human-Machine Interfaces (HMI), Augmented Reality (AR) and robotics [4,8,27]. These applications require accurate and stable reconstructions. Robust, sequential and real-time reconstruction methods are thus a priority in most of these applications. In addition, new methods must be able to deal with real conditions such as the data association problem between images, which includes outliers and missing data.

## 2. Related Works

This section includes a summary and discussion of the most recent methods for 3D reconstruction from images. Section 2.1 discusses the SfM methods. The SfT and NRSfM methods are presented in Section 2.2 and Section 2.3 respectively.

### 2.1. Structure-From-Motion

Structure-from-motion can be defined as the problem of jointly inferring the 3D geometry of the scene and camera motion using images as inputs. The rigidity of the scene is a prior condition for SfM. The geometry of multiple views of a rigid scene has been known for centuries, and the basic results for solving SfM are well known in photogrammetry and computer vision [1]. Modern SfM methods cope with large sequences from both uncalibrated and calibrated cameras.

Sequential approaches in SfM are very important in robotics and were mainly developed as solutions to the visual Simultaneous Localization and Mapping (vSLAM) problem, where the robot pose and a map of the 3D environment are sequentially obtained from a camera mounted on a mobile robot. Given the improvements of modern SfM algorithms, vision-based SLAM quickly became predominant in robotics and is usually fused with other sensors installed in the robot.

One of the first monocular vSLAM systems was proposed in [4]. The SLAM problem was posed as a sequential Bayesian inference problem, using the Extended Kalman Filter (EKF) as the inference core and a sparse set of salient feature tracks as image observations. This method was implemented in real time using a low cost hardware and was suitable to reconstruct small- to medium-sized environments.

Years later, another sparse SLAM method was proposed, based on sequential Bundle Adjustment (BA) and known as Parallel Tracking and Mapping (PTAM) [8]. This method showed very accurate and stable pose estimation and reconstructions, suitable for AR applications. It remarkably improved vSLAM methods based on statistical filtering. PTAM heavily influenced modern SLAM methods and changed the SLAM processing paradigm from pure sequential to a parallel mapping and tracking algorithm.

Recently, dense SfM methods have been proposed, such as Dense Tracking and Mapping (DTAM) [9], further extended to depth sensors, such as Kinect fusion [28]. SfM methods fail to recover the shape of non-rigid objects. This motivates the methods discussed next.

### 2.2. Shape-From-Template

Shape-from-Template (SfT) methods estimate the 3D shape of a non-rigid object from a single image and a template of the object. A template consists of the reference shape of the object, a texture map and a deformation model. The template can be computed from samples or even generated by other reconstruction algorithms from a training sequence. The objective of SfT is to find the deformed 3D shape given a single image or a sequence of images and the object template. There are two main categories of SfT methods based on the deformation model: statistics-based and physics-based.

Regarding the first group, the most commonly-known algorithm is represented by the Active Appearance Models (AAM), which are widely used for the tracking of the human face. The first AAM method was introduced by [29]. In the context of non-rigid reconstructions, the AAM evolved into the so-called 3D Morphable Models (3DMM) [30]. A revisiting example of these models including an extensive learning and their application over a database of 10,000 faces is studied in [31].

AAMs and 3DMMs reconstruct objects that belong to a specific category whose deformations and appearance are low dimensional. This makes them very suitable for 3D reconstruction of face gestures. They need large labeled databases for a proper training of the shape and appearance models. Hand-made labeled databases are prone to errors, not only due to the “human factor”, but also as different people do this task differently. In [32], the authors take advantage of some of the approaches and dense correspondences to infer the point correspondences and predict the labeling.

Most of remaining SfT methods are based on physics-based deformation priors and in particular the isometric model [17,33,34]. The work in [17] describes the problem as a Partial Differential Equations (PDE) system and proves that imposing the isometry prior makes SfT a well-posed problem. Methods in SfT can be divided into local solutions, mainly based on solutions of a PDE system, and global solutions, based on convex relaxations of isometry [18,19].

The registration between the template and the model is needed in SfT. In most of the SfT papers, the registration is assumed to be known or obtained with some automatic methods, like in [35], based on graph matching, or dense optical flow methods, such as [36]. These methods are however not suitable for real-time applications.

### 2.3. Non-Rigid Structure from Motion

In NRSfM, the objective is to recover the 3D shape of an object under deformations from a sequence of images. Each image captures the combination of rigid motion and shape change in the object. Thus, rigid SfM is not applicable in this case. NRSfM is ill-posed, unless priors on the possible deformations are imposed. According to the deformation prior, existing NRSfM methods can be divided into two main groups: (i) physics-based and (ii) statistics-based methods.

In physics-based models, the deformation model is taken from the field of continuum mechanics, and it models how materials behave under the action of forces. The most popular models used in this category are the isometric model [14,15,16,34,37] and the elastic (linear and non-linear) model [23,38,39,40]. The isometric model has been thoroughly studied, and [15] proved that isometric NRSfM is a well-posed problem. However, isometric priors are not accurate to describe deformations suffered by soft materials. Elastic models have been proposed in [23,38,39,40], using the Finite Element Modeling (FEM) approximations of linear and non-linear elastic materials. They estimate both camera pose and the 3D shape of deformable objects from monocular scenes in real time. These methods require in general object-dependent physical parameters, except for [39] in which the deformation modes are estimated sequentially. Besides, existing NRSfM is not well posed with elastic models as it has been empirically confirmed that these methods require additional constraints to limit ambiguities.

Some methods assume that the shape of the object is composed of pieces, following simple local deformation models such as rigid or quadratic [41,42]. The main challenge in these approaches is how to split the data into a set of models automatically and how to preserve global coherence of the model.

In statistics-based approaches, the object shape space is assumed to be low-dimensional and is represented as the weighted sum of a set of basis shapes. This idea was first proposed by [10], and it is based on the assumption that the shape matrix is low-rank. This prior has been studied by many researchers in the NRSfM literature [11,13,43,44]. It has proven to be successful in many real-world scenarios such as in the gestures of the human face. NRSfM based on the low-rank model is ill-posed, as the solution space is very ambiguous. Some methods have added priors, such as temporal smoothness or spacial smoothness [11], physical priors such as limiting stretching or extension in the surface [33,34] or the use of trajectory bases [45]/shape-trajectory bases [20], which constrain the trajectory of point tracks along the sequence.

Most of aforementioned methods are based on tracking a sparse set of image correspondences, recovering a sparse 3D model of the object. Dense NRSfM approaches have been recently investigated. The first one is [43], which performs dense optical flow combined with low-rank modeling and local smoothness priors. In [46], the segmentation and reconstruction of local rigid models is proposed. On the other hand, Kinect fusion was further extended to handle deformable objects in dynamic fusion [47] for depth cameras. However, these approaches need the use of specialized hardware (GPU-based) to be run in real time.

The majority of the NRSfM methods use orthographic projection. This is an advantage for statistics-based algorithms [10,11,12,20,21,44], as it allows one to pose NRSfM as a low-rank matrix factorization problem, which counts with global and efficient solvers. However, the orthographic camera can be very inaccurate for images taken under certain imaging conditions. It also includes ambiguities in the reconstructions due to the convex/concave shape flips and the depth ambiguity problem. Some NRSfM methods include the perspective camera such as [22,48,49,50], which is a more accurate model. However, it makes the problem harder to solve.

### 2.4. Proposal

This paper proposes a sequential solution to SfT able to be run in real time (e.g., 15–30 frames per second) using a low-cost hardware based on a CPU. Our algorithm is efficient and very suitable for embedded systems. These features are available in late SfM methods [51], but not in the context of non-rigid reconstruction.

We take PTAM, a state-of-the-art sequential real-time SfM engine, and we upgrade the tracking thread to solve SfT using a low-rank shape basis model. As in PTAM, we use a calibrated perspective camera model. As previously mentioned, the perspective camera is an accurate model and does not suffer from some ambiguities present in the orthographic model, widely used for statistics-based non-rigid reconstruction methods.

Most of the current state-of-the-art non-rigid reconstruction algorithms do not compute the tracking and data association, whereas in a real applications, this is one of the most important challenges to face. Our solution deals with data association in real time. This paper extends the work published by the authors in [52] where a preliminary version of our tracking algorithm was presented. In this work, an improved version is implemented, and a deeper analysis is carried out. We hereinafter refer to this method as the IROS-based approach.

This paper is organized as follows: The description of our method is given in Section 3. In Section 3.1, we describe the data-association model, and in Section 3.2 and Section 3.3, we describe the feature matching method and the camera motion models, respectively. We present our shape inference method in Section 3.4 and Section 3.5. In Section 4, a summary of the results is described. Finally, Section 5 presents the main conclusions of the paper.

## 3. Algorithm Description

**Figure 1:**
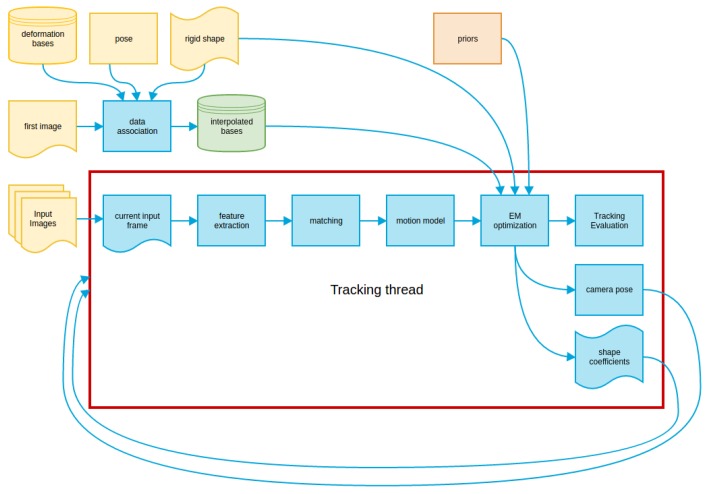
Tracking thread flowchart. The main modules of our tracking thread are included inside the red box. The yellow modules represent inputs; the orange modules are configurations (priors); the green ones are estimations from the input data; and finally, the blue ones are implemented tasks.

Our algorithm estimates the camera pose and the shape of the object in each frame of a video sequence. Image cues are based on tracking a sparse set of salient features over the images. To that end, it needs an initial estimation of the object’s rigid shape, the camera pose and the object shape basis. We refer to this method as the tracking thread, as it is based on the tracking thread of PTAM. A flowchart of the tracking thread and the initialization process is presented in Figure 1.

To model the deformations, we use a linear shape basis model [10]. The shape of the object can be expressed as the weighted sum of a finite number of shape vectors:

(1) $$S_{NR}\left(f\right)=S_{R}+\sum_{k=1}^{K}L_{k}\left(f\right)B_{k}$$

where *f* represents the frame number, *K* is the number of shapes given in the model, $S_{NR}\left(f\right)\in R^{3\times P}$ is the computed shape for frame *f* and *P* represents the number of points of the shape. $S_{R}\in R^{3\times P}$ is the rigid shape average. $L\left(f\right)\in R^{K}$ are the shape coefficients, and $B\in R^{3K\times P}$ are the basis shapes, which were previously known.

The transformation from world to camera coordinates for each point is given by:

(2) $$X_{i}={\left(\begin{array}{ccc} x_{i} & y_{i} & z_{i} \end{array}\right)}^{T}=\left[R|T\right]\times S_{NR_{i}}$$

where the camera rotation is given by $R\in R^{3\times 3}$ and the camera translation is represented by $T\in R^{3}$. Each 3D point in camera coordinates is projected using the perspective camera model:

(3) $$U_{i}^{S}=\left(\begin{array}{c} u_{i}\\ v_{i} \end{array}\right)=\left(\begin{array}{c} u_{0}\\ v_{0} \end{array}\right)+\alpha \left(\begin{array}{cc} f_{u} & 0\\ 0 & f_{v} \end{array}\right)\left(\begin{array}{c} x_{i}/z_{i}\\ y_{i}/z_{i} \end{array}\right)$$

where $U^{S}\in R^{2\times P}$ represents the set of shape points, ${\left(u_{0},v_{0}\right)}^{T}$ are the camera center coordinates, $\left(f_{u},f_{v}\right)$ is the focal length and α is the radial distortion function. These parameters are estimated in an offline calibration process described in [8].

### 3.1. Measurement Model/Data Association

**Figure 2:**
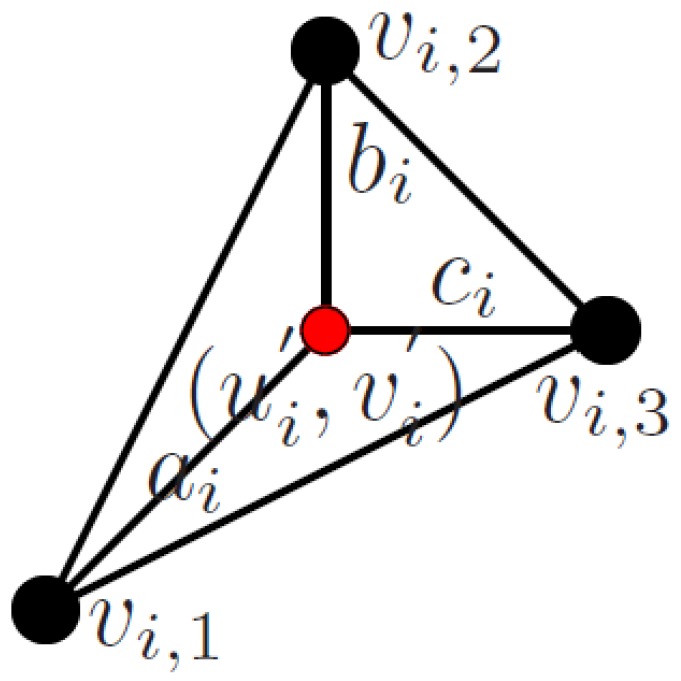
Data association from detected points (red) with model points in mesh (black).Tracking data association

The vertices of the shape model $S_{NR}$ do not match in general with the points detected in the image using feature detectors described in Section 3.2.2. To establish a link between the shape model and the measurements, we use a triangular mesh model. The mesh is estimated by using the Delaunay triangulation on the projected coordinates of our shape model ($S_{NR}$) in the first frame of the sequence $U_{0}^{S}$. This gives a mesh of connected vertices $v_{i}$. This mesh is fixed for the rest of the sequence. Given a point detected in the image $U_{i}^{{}^{\prime }}={\left(u_{i}^{{}^{\prime }}v_{i}^{{}^{\prime }}\right)}^{T}$, we find the mesh triangle it belongs to (the three vertices of the triangle $v_{i,1\ldots 3}$) and compute the barycentric coordinates $\left(a_{i},b_{i},c_{i}\right)$, following [53]:

(4) $$\left(\begin{array}{c} a_{i}\\ b_{i}\\ c_{i} \end{array}\right)=pinv\left(\begin{array}{ccc} v_{i,1x} & v_{i,2x} & v_{i,3x}\\ v_{i,1y} & v_{i,2y} & v_{i,3y} \end{array}\right)\times \left(\begin{array}{c} u_{i}^{{}^{\prime }}\\ v_{i}^{{}^{\prime }} \end{array}\right)$$

where pinv(·) is the matrix pseudo-inverse operator. An example of the data association is represented in Figure 2, where the projected mesh vertices of the triangle are represented as black points, and the detected point is represented as a red point inside the triangle. The barycentric coordinates are displayed as edges connecting the detected point and the vertices of the triangle.

We can define a new deformation basis $B^{{}^{\prime }}$, adapted to the detected points and defined in world coordinates, by using the barycentric coordinates. The 3D position of the interpolated model point $X_{i}^{{}^{\prime }}$ that corresponds to the 2D feature $U_{i}^{{}^{\prime }}$ relates to the model basis shapes as:

(5) $$X_{i}^{{}^{\prime }}=a_{i}X_{v1}+b_{i}X_{v2}+c_{i}X_{v3}$$

(6) $$X_{i}^{{}^{\prime }}=\sum_{k}L_{k}\left(a_{i}B_{v1k}+b_{i}B_{v2k}+c_{i}B_{v3k}\right)=\sum_{k}L_{k}B_{ik}^{{}^{\prime }}$$

where $X_{i}^{{}^{\prime }}$ is the interpolated point corresponding to the detected point $U_{i}^{{}^{\prime }}$ and the triangulation defined by the barycentric coordinates $\left(a_{i},b_{i},c_{i}\right)$ and $X_{v1},X_{v2},X_{v3}$, which are the 3D coordinates of the 2D associated coordinates vertices $\left(v_{i,1},v_{i,2},v_{i,3}\right)$ and $\left(B_{v1k},B_{v2k},B_{v3k}\right)$, are k-th bases of each of the vertices of the triangle. Equation (6) shows the duality of working with model or detected points and interpolated 3D points, once the data association is set.

### 3.2. Feature Matching

We detect and match point features between the reference image and the rest of the images in the video sequence. The quality of feature detection and matching is crucial for obtaining accurate reconstructions. To achieve real-time performance, we face a trade-off between the quality of matching and the complexity of the existing feature detection and matching methods.

Two different feature detection and matching methods are studied. The first one is based on PTAM, which uses the FAST detector and was presented by the authors in [52]. This is considered as the baseline for the comparison. A second approach is based on descriptor matching and uses different state-of-the-art visual detectors and descriptors.

#### 3.2.1. PTAM-Based Matching Approach

Hereinafter, we assume that the points detected in the image ($U^{{}^{\prime }}$), e.g., using the FAST [54] detector, correspond to the projection of the points described by the interpolated bases $B^{{}^{\prime }}$. This saves computation time and makes equations simpler.

We use the same process described in [8] to track points in the video sequence. However, due to shape deformations, the appearance in a local region around each point can suffer variations. We thus propose the following adaptations:

- Affine warping described in [8] is kept to handle points whose local appearance is affected by a rigid motion.
- If the deformation causes significant changes in the appearance, affine warping is expected to fail. Then, we search features in a coarse-to-fine hierarchical correlation-based approach. To discard false positives, a married matching is applied in the lowest level of the pyramid in which the feature has been found. In case the correlation is too low or the distance is too high, the matching is considered not found. For a deeper explanation, we refer the readers to our publication in [52].

This algorithm could fail for points with a highly deformed texture or deformations that imply large movements between images. Rejected points are considered as outliers and are discarded. This prevents tracking from degenerating in the following frames.

#### 3.2.2. Descriptor-Based Matching Approach

The detection and matching layer of the IROS-based approach is substituted by descriptor-based features implemented in OpenCV [55]. Some of them are directly available, like KAZE [56], AKAZE [57], ORB [58] and BRISK [59], and others are not directly included, but can be accessed as third party software for research purposes, like SIFT [60] and SURF [61]. Binary descriptors, such as ORB, AKAZE and BRISK, are preferred for a real-time implementation as they are faster.

Once the features are detected and described, we match them across frames. Several matching algorithms are used such as: brute force, L1, L2 and Hamming distance. These techniques are prone to establish incorrect matches.

Matches are searched inside a circular area defined in the image domain. The criterion we follow is to select matches of the minimum descriptor distance that do not violate the maximum radius condition. The radius parameter is configurable; thus, it can be tuned if the number of tracking failures is high.

Feature descriptors, such as SIFT or KAZE, are not completely affine invariant and, thus, can fail with high deformations. Specialized feature descriptors exist in the literature for deformable registration, such as in [62,63]. We assume in this paper that strong deformations are not present in local patches, so these options are not considered.

### 3.3. Motion Modeling

A linear decaying motion model is used to improve camera tracking convergence. The pose is updated using the following motion model:

(7) $$\begin{array}{c} vel_{t}=\beta /2\left(vel_{t-1}+\mu \right)\\ {\hat{\left[R|T\right]}}^{t}=exp\left(vel_{t}\Delta t\right){\left[R|T\right]}^{t-1} \end{array}$$

In order to apply the equation, the camera speed $vel_{t}$, between frames t−1 and *t*, is computed by applying the ESMhomography method proposed in [64]. β is the factor that balances the influence of the estimated camera speed on the camera motion update vector μ. This motion model was proposed in the original PTAM algorithm [8], where the shape was assumed to be rigid. We adapt it by using the updated shape obtained in the last frame, as will be explained in Section 3.4.

### 3.4. EM Optimization

We present in this section the shape-inference algorithm that estimates the shape deformation coefficients and the camera pose of the current frame. We use an optimization approach where we minimize the reprojection error between the detected points and the deformed shape projected using the perspective camera. This is a non-convex cost that cannot be solved in closed form. Previous approaches, like [11,24,65,66], implement Expectation-Maximization (EM) algorithms to split the optimization of a similar cost function into two parts: deformation weights, keeping the camera parameters fixed and vice versa. This strategy is easy to implement and has faster convergence time, contributing to the real-time constraint. We have adapted this method to our problem.

The Maximum Likelihood Estimation (MLE) function is defined as,

(8) $$E_{data}=f_{MLE}\left(R,T,L\right)\propto \sum_{i}{\left|U_{i}^{{}^{\prime }}-proj\left(X_{i}^{{}^{\prime }}\right)\right|}^{2}$$

where $E_{data}$ represents the 2D reprojection error. It is minimized w.r.t. the following two sets of parameters:

- Pose parameters (6 DoF): rotation *R* and translation *T* defined by the vector of parameters $\mu =\left[\phi_{x},\phi_{y},\phi_{z},t_{x},t_{y},t_{z}\right]$.
- Deformation parameters (*K* DoF): the set of *K* shape deformation coefficients *L*.

Both sets of parameters are grouped in the state vector $\theta =\left[\begin{array}{cc} \mu & L \end{array}\right]$, which has 6+K DoF. The matrices $B^{{}^{\prime }}$ and $S_{R}$ are assumed to be fixed during the sequence. A maximum of ten iterations is used for each frame in order to estimate camera pose and deformation coefficients by using the alternation given in the EM algorithm. The actual iterations are obtained checking the RMS reprojection error according to Equation (9). Two consecutive increases on the error or low error reductions makes the minimization stop. Among all the estimations, the best one is kept as the solution for the current frame.

(9) $$e_{RMS}=\sqrt{\frac{\sum_{i}{∥e_{i}∥}^{2}}{\sum_{i}{∥U_{i}^{{}^{\prime }}∥}^{2}}}=\sqrt{\frac{\sum_{i}{∥U_{i}^{{}^{\prime }}-proj\left(X_{i}^{{}^{\prime }}\right)∥}^{2}}{\sum_{i}{∥U_{i}^{{}^{\prime }}∥}^{2}}}$$

#### 3.4.1. E-Step: Deformation Estimation

The main objective in this step is to minimize $f_{MLE}$ as a function of the deformation parameters *L*, fixing the pose parameters μ. We denote by ${\hat{\left[R|T\right]}}^{t-1}$ the current camera pose, derived from the previous M-step, and we recall that $S_{R}$ and $B^{{}^{\prime }}$ remain fixed along the process. Given:

(10) $$e_{i}^{L}=\left(\begin{array}{c} u_{i}^{{}^{\prime }}\\ v_{i}^{{}^{\prime }} \end{array}\right)-proj\left({\hat{\left[R|T\right]}}^{t}\sum_{k}L_{k}^{t-1}B_{k,i}^{{}^{\prime }}\right)$$

where $e_{i}^{L}$ represents the re-projection error of the *i*-th point in the current frame and $L^{t-1}$ is the current value for the deformation weights. An M-estimator using the Tukey bi-weight function [67] is used to help with outlier rejection [8]. The computed weights depend on each point reprojection error. Only matched points are processed. In order to estimate the shape coefficients, we start undistorting the projections, from Equation (3). The orthographic projection has as the main advantage that the factorization can be directly applied to the tracking matrix to obtain the coefficients, as the projection of the sum is the sum of the projections. However, for the perspective projection case, this does not hold, as it depends on the depth. We use Equations (2) and (3) to obtain the following result:

(11) $$\left(\begin{array}{c} \lambda u\\ \lambda v\\ \lambda \end{array}\right)=\left(\begin{array}{c} f_{u}\left(\sum_{k}L_{k}{\vec{r}}_{x}B_{k}^{{}^{\prime }}+t_{x}\right)+u_{0}\lambda \\ f_{v}\left(\sum_{k}L_{k}{\vec{r}}_{y}B_{k}^{{}^{\prime }}+t_{y}\right)+v_{0}\lambda \\ \sum_{k}L_{k}{\vec{r}}_{z}B_{k}^{{}^{\prime }}+t_{z} \end{array}\right)$$

where λ is the perspective projection scale, $R={\left(\begin{array}{ccc} {\vec{r}}_{x} & {\vec{r}}_{y} & {\vec{r}}_{z} \end{array}\right)}^{T}$ is the rotation matrix decomposed in vectors and $T={\left(\begin{array}{ccc} t_{x} & t_{y} & t_{z} \end{array}\right)}^{T}$ is the translation vector expressed in its three components.

Grouping the terms in $L_{k}$ on one side and writing the equivalent system in a Linear Least Squares (LLS) form Ax=C, where x=L, $\Delta u_{p}=u_{p}-u_{0}$, and $\Delta v_{p}=v_{p}-v_{0}$, we obtain:

(12) $$A=\left(\begin{array}{ccc} \left(\Delta u_{1}{\vec{r}}_{z}-f_{u}{\vec{r}}_{x}\right)B_{11}^{{}^{\prime }} & \cdots & \left(\Delta u_{1}{\vec{r}}_{z}-f_{u}{\vec{r}}_{x}\right)B_{1K}^{{}^{\prime }}\\ \left(\Delta v_{1}{\vec{r}}_{z}-f_{v}{\vec{r}}_{y}\right)B_{11}^{{}^{\prime }} & \cdots & \left(\Delta v_{1}{\vec{r}}_{z}-f_{v}{\vec{r}}_{y}\right)B_{1K}^{{}^{\prime }}\\ \cdots & \cdots & \cdots \\ \left(\Delta u_{P}{\vec{r}}_{z}-f_{u}{\vec{r}}_{x}\right)B_{P1}^{{}^{\prime }} & \cdots & \left(\Delta u_{P}{\vec{r}}_{z}-f_{u}{\vec{r}}_{x}\right)B_{PK}^{{}^{\prime }}\\ \left(\Delta v_{P}{\vec{r}}_{z}-f_{v}{\vec{r}}_{y}\right)B_{P1}^{{}^{\prime }} & \cdots & \left(\Delta v_{P}{\vec{r}}_{z}-f_{v}{\vec{r}}_{y}\right)B_{PK}^{{}^{\prime }} \end{array}\right)$$

(13) $$C=\left(\begin{array}{c} f_{u}t_{x}-t_{z}\Delta u_{1}\\ f_{v}t_{y}-t_{z}\Delta v_{1}\\ \vdots \\ f_{u}t_{x}-t_{z}\Delta u_{P}\\ f_{v}t_{y}-t_{z}\Delta v_{P} \end{array}\right)$$

If an average shape is given in the initialization ($S_{R}$), it must be taken out from the estimation. If this is the case, it can be described as a function of the basis shapes:

(14) $$S_{R}=\sum_{k}L_{R_{k}}B_{k}$$

Then, in Equation (14), the average rigid shape $S_{R}$ is described as a function of an additional set of coefficients, the rigid coefficients $L_{R}\in R^{K}$. Doing $L_{NR}=L-L_{R}$ removes the rigid shape influence from the estimations.

If the previous condition does not hold, the rigid shape must be introduced in the deduction of the matrices *A* and *C*, as the 3D model and then the projection equations need to be adapted. The *A* matrix remains the same as in Equation (12), but *C* gets more complex, as indicated in Equation (15).

(15) $$C=\left(\begin{array}{c} f_{u}t_{x}-t_{z}\Delta u_{1}+S_{R,1}\left(f_{u}\vec{r_{x}}-\vec{r_{z}}\Delta u_{1}\right)\\ f_{v}t_{y}-t_{z}\Delta v_{1}+S_{R,1}\left(f_{v}\vec{r_{y}}-\vec{r_{z}}\Delta v_{1}\right)\\ \vdots \\ f_{u}t_{x}-t_{z}\Delta u_{P}+S_{R,P}\left(f_{u}\vec{r_{x}}-\vec{r_{z}}\Delta u_{P}\right)\\ f_{v}t_{y}-t_{z}\Delta v_{P}+S_{R,P}\left(f_{v}\vec{r_{y}}-\vec{r_{z}}\Delta v_{P}\right) \end{array}\right)$$

#### 3.4.2. M-Step, Pose Estimation

The goal in this minimization step is to compute the camera pose. This is done by maximizing the likelihood of the observed data, as shown in Equation (8). In this step, the 3D shape is kept fixed, minimizing the following reprojection error expression:

(16) $$e_{i}^{\mu }=\left(\begin{array}{c} u_{i}^{{}^{\prime }}\\ v_{i}^{{}^{\prime }} \end{array}\right)-proj\left(\exp\left(\mu^{t}\right){\hat{\left[R|T\right]}}^{t}\sum_{k}L_{k}^{t}B_{k}^{{}^{\prime }}\right)$$

Equations (10) and (16) are similar, but in this case, *L* is known from the last E-step, and μ (the camera update) is unknown. We compute μ as in [8]. Similarly to the E-step, the missing correspondences are taken out from the pose estimation on the current frame.

Since the pose parameters are not linear, the estimation cannot be done in closed form. Instead, it is based on several steps of gradient descent, for which the pose update w.r.t. the error must be obtained. To that end, Equation (3) is decomposed into several matrices to facilitate the derivation of the expression using the chain rule.

After several steps, the following expression is reached:

(17) $${\left.\frac{\partial e_{i}^{\mu }}{\partial \mu_{p}}\right|}_{2\times 6}={\left.J_{cam}\right|}_{2\times 2}\left({\left.J_{D_{i}}\right|}_{2\times 3}\begin{array}{c} 0\\ 0 \end{array}\right){\left.G_{p}\right|}_{4\times 4,p=1\ldots 6}{\left.E_{CW}\right|}_{4x4}{\left.X_{W}\right|}_{4\times 1}$$

where p∈1…6 denotes the index in the pose parameters, $G_{p}$ the generation matrix of the SE(3) Lie group and $E_{CW}$ is the pose matrix composed by the rotation matrix *R* and the translation vector *T*. ${\left.J_{cam}\right|}_{2\times 2}$ is defined as follows:

(18) $${\left.J_{cam}\right|}_{2\times 2}={\left.J_{A}\right|}_{2\times 2}{\left.J_{C}\right|}_{2\times 2}$$

where $J_{A}$ is the derivative w.r.t the 3D point in camera coordinates of Equation (3) (removing distortion) and $J_{C}$ is related to the derivative of the distortion part of the calibration matrix w.r.t the 3D point in camera coordinates:

(19) $$J_{A}=\left(\begin{array}{cc} f_{u} & 0\\ 0 & f_{v} \end{array}\right)$$

(20) $$J_{C}=\left(\begin{array}{cc} \hat{B} & 0\\ 0 & \hat{B} \end{array}\right)+\left(\begin{array}{c} C_{1}\\ C_{2} \end{array}\right)J_{\hat{B}}$$

$\hat{B}=\frac{\tilde{r}}{r}$ with $r=\sqrt{C_{1}^{2}+C_{2}^{2}}$, $\tilde{r}=r-\beta_{1}r^{3}-\beta_{2}r^{5}$, $C_{1}=\frac{x_{c}}{z_{c}}$, $C_{2}=\frac{y_{c}}{z_{c}}$, $\left(x_{c},y_{c},z_{c}\right)$ are the 3D coordinates of the points in camera reference system, and:

(21) $$J_{\hat{B}}={\left(\begin{array}{c} -2\beta_{1}C_{1}-\beta_{2}\left(4C_{1}^{3}+4C_{1}C_{2}^{2}\right)\\ -2\beta_{1}C_{2}-\beta_{2}\left(4C_{2}^{3}+4C_{2}C_{1}^{2}\right) \end{array}\right)}^{T}$$

where $\beta_{1}$ and $\beta_{2}$ denote the distortion coefficients computed from camera calibration.

Lastly, $J_{D}$ is the derivative of the point w.r.t. its coordinates in the camera reference:

(22) $$J_{D}=\left(\begin{array}{ccc} \frac{1}{z_{c}} & 0 & \frac{-x_{c}}{z_{c}^{2}}\\ 0 & \frac{1}{z_{c}} & \frac{-y_{c}}{z_{c}^{2}} \end{array}\right)$$

The Jacobian of Equation (17) is computed for each point $X_{i}^{{}^{\prime }}$ in world coordinates ($X_{W}$) and then inserted into a WLS minimizer together with an M-estimator, for outlier rejection.

Once the update μ for the pose is obtained, as the pose belongs to the SE(3) Lie group, the pose is then updated as:

(23) $$E_{CW}^{{}^{\prime }}=\exp\left(\mu \right)E_{CW}$$

### 3.5. Priors

In order to improve the method, we include regularization and smoothing priors in the optimization process. These tracking priors allow us to obtain better 3D error by smoothing the pose and shape solutions over the sequence.

After a revision of the related works, we found out that the most suitable priors for this tracking problem are temporal and shape smoothness, as they are easily adapted to a least squares minimization scheme in the E-step.

Some more complex priors were considered, such as the isometric prior. We discarded them as they generate non-convex costs, which do not fit well in our EM approach. Isometry is also a strong prior that cannot be used for some objects that undergo elastic deformations.

The consequence of including the priors is the modification of the error function to be minimized. Instead of relying only on the 2D reprojection error, the cost function takes into account the following terms:

(24) $$E=E_{data}+\rho_{temp}E_{temp}+\rho_{shape}E_{shape}$$

$E_{data}$ is the 2D reprojection error described in Equation (8):

(25) $$E_{data}=\sum_{i=1}^{P}∥U_{i}^{{}^{\prime }}-proj\left(X_{i}^{{}^{\prime }}\right)∥$$

$E_{temp}$ imposes temporal smoothness:

(26) $$E_{temp}=\sum_{i=1}^{P}∥X_{i}^{{}^{\prime }}\left(f\right)-X_{i}^{{}^{\prime }}\left(f-1\right)∥$$

and $E_{shape}$ is an extra restriction of the shape where deviations from the reference shape are penalized:

(27) $$E_{shape}=\sum_{i=1}^{P}∥X_{i}\left(f\right)-\hat{X_{i}}\left(f\right)∥$$

where $\rho_{temp}$ is the weight for temporal smoothness and $\rho_{shape}$ is the weight for shape smoothness.

$E_{temp}$ imposes a temporal dependence between the current 3D shape $X_{i}^{{}^{\prime }}\left(f\right)$ and the previous $X_{i}\left(f-1\right)$. Decomposing $X_{i}^{{}^{\prime }}\left(f\right)=B_{i}^{{}^{\prime }}L\left(f\right)$, the expression could be directly solved as it can be put as a least squares expression function of *L*.

$E_{shape}$ forces the current shape $X_{i}\left(f\right)$ to be closer to the shape ${\hat{X}}_{i}\left(f\right)$, obtained by predicting each node of the mesh using its neighbors:

(28) $${\hat{X}}_{i}\left(f\right)=\sum_{k,m,n\in ℵ\left({\hat{X}}_{i}\right)}\alpha_{i}X_{k}+\beta_{i}X_{l}+\gamma_{i}X_{m}$$

where $\alpha_{i}$, $\beta_{i}$ and $\gamma_{i}$ are the barycentric coordinates of vertex *i* in the mesh with respect to three neighboring vertices $X_{k}$, $X_{l}$ and $X_{m}$. Note that the barycentric coordinates are computed using the reference shape. This term imposes spatial smoothness by penalizing shapes with points that do not match with their neighbors. This equation can be drawn as a least square expression with Las a free parameter, so Equation (24) can be optimized in the E-step.

## 4. Results

Firstly, a general overview of the performance metrics is described followed by a revision of the different datasets used for the assessment of the algorithm and a detailed report of the results for each of the sequences.

### 4.1. Performance Metrics

This section presents the metrics used to evaluate the performance of our algorithm for different test sequences (motion captured or synthetically generated). After a sequence is processed by the algorithm, the output results are post-processed to compare the performance of the algorithm using the ground truth. The way that the sequences are adapted to our system is also explained.

We evaluate reconstruction error using the following indicators:

- 2D error:
  (29) $$2D\;err\;\left(px\right)=\frac{1}{F}\sum_{f=1}^{F}\frac{\sqrt{\sum_{p}{∥x_{est}\left(f,p\right)-x_{gt}\left(f,p\right)∥}_{2}^{2}}}{\sqrt{\sum_{p}{∥x_{gt}\left(f,p\right)∥}_{2}^{2}}}max\left(x_{gt}\right)$$
  where *F* is the number of frames of the sequences, *P* is the number of points, $x_{est}=\left(u_{est},v_{est}\right)$ are the re-projected features on the image and $x_{gt}=\left(u_{gt},v_{gt}\right)$ represent the image coordinates of the ground-truth points.
- 3D error:
  (30) $$3D\;err\left(\%\right)=\frac{1}{F}\sum_{f=1}^{F}\frac{\sqrt{\sum_{p}{∥S_{est}\left(f,p\right)-S_{gt}\left(f,p\right)∥}_{2}^{2}}}{\sqrt{\sum_{p}{∥S_{gt}\left(f,p\right)∥}_{2}^{2}}}\times 100$$
  $S_{est}=\left(x_{est},y_{est},z_{est}\right)$ is the estimated shape and $S_{gt}=\left(x_{gt},y_{gt},z_{gt}\right)$ the ground truth shape.

The 2D reprojection error and the 3D reconstruction error were defined in [21]. When applied to an entire sequence, the error indicators are either averaged or presented individually for each frame. The former is useful to analyze the evolution of the error over time.

Regarding the 3D error, a previous Procrustes analysis [68] is carried out before applying Equation (30). It rotates and scales the shapes to align them w.r.t. the ground-truth. This is a common procedure in the state-of-the-art works, such as in [12,21]. Unless the opposite is explicitly indicated, this alignment is always applied before computing 3D errors.

### 4.2. Setup

In order to run the algorithm, we need a set of basis shapes, a rigid shape and an initial pose. These are estimated from the ground truth data as follows:

The rigid shape $S_{R}$ is computed by extracting the average of the set of shapes used to obtain the bases. The initial pose is retrieved from the projection on the initial frame. We use PCA to retrieve the set of shape bases. The rigid shape is previously subtracted from the shapes, and no Procrustes alignment is applied before factorization. The basis shapes are then computed using PCA from this expression:

(31) $$S-S_{R}=\left(\begin{array}{ccccccc} x_{1,1} & y_{1,1} & z_{1,1} & \cdots & x_{1,P} & y_{1,P} & z_{1,P}\\ \vdots & \vdots & \vdots & \vdots & \vdots & \vdots & \vdots \\ x_{F,1} & y_{F,1} & z_{F,1} & \cdots & x_{F,P} & y_{F,P} & z_{F,P} \end{array}\right)=UDV$$

After PCA is computed from this matrix, the bases ($B=\sqrt{D}V$) are obtained, but only the *K* most relevant components are taken. The number of bases used for all the experiments are at least K=15, unless the opposite is indicated. We always ensure that the value of *K* represents at least 85% of the total deformation energy obtained from the PCA decomposition following the expressions in Equations (32) and (33). A higher number of bases can lead to over-fitting when dealing with real data, not to mention the memory and computational costs of handling a large number of bases. These are the main reasons why a fixed value (unless specified) of bases is set.

(32) $$D_{normalized}=\frac{diag(D)}{\sum diag(D)}$$

(33) $$D_{cumulated}\left[i\right]=\sum_{j=0}^{i}D_{normalized}\left[j\right]\;\forall i$$

It must be noted that no Procrustes alignment is applied before the SVD factorization.

To handle the data from motion captured datasets, without any kind of visual information, a special version of the tracking thread is developed, to accept as input text files containing the point projections for each frame, the 3D bases, the pose initialization, the visibility masks, etc. The ground truth 3D points are projected using the perspective camera (Equation (3)), choosing a convenient pose to see all the sequence points over time.

To perform a thorough comparison and to simulate real tracking conditions for these datasets, the following sets of outlier percentage values $outl=\left[0,5,10,20,30,40\right]$% and noise strength values $\sigma =\left[0,1,2,3,4\right]$ are added to the set of projected points. This is similar to the evaluation proposed in [53]. With this setup, an initial evaluation is performed to check the tracking robustness under these conditions. The added noise follows a normal distribution $N\left(0,\sigma^{2}\right)$. A percentage of the image points is marked as outliers. For those points both spacial directions are randomly deviated 20 pixels. Point visibility is also introduced as a variable to analyze algorithm robustness. To that end, randomly distributed visibility masks in an increasing percentage are generated.

All the experiments are computed using an i7 laptop with 8 GB RAM and eight virtual core processor at 2.4 GHz. The algorithm is implemented in C++ and runs on Ubuntu Linux, using PTAM-derived libraries, OpenMP, CGAL and OpenCV. If it is not specified, the tests for the methods of [11,12,20,21] are executed with the default parameters given by the authors. We set the motion movement constant β to 0.9; the radius limit of the IROS-based feature detection is 30 px; and the one for the rest of the matching algorithms is 70 px.

A general overview of the algorithm performance is presented with two motion capture datasets. We start evaluating the tracking with the assumption that the matching is perfect. After that, the results are re-evaluated for tracking under visibility degradation, noise, outliers and varying the number of basis shapes. Smoothing priors are not included in these experiments.

For real images, we analyze the performance by testing some matching methods, varying the number of basis shapes, and finally, we analyze the performance using the smoothing priors. Using real images implies that missing data, outliers and noise are already present in the data.

### 4.3. Flag Sequence

A motion captured flag bending with the wind is represented in the flag sequence. The deformations are so significant that they make this dataset challenging. Moreover, the amount of points in the sequence (540 in 450 frames projected on 640 × 480 images) is significantly higher than in other synthetic datasets used in the state-of-the-art, which implies a challenge to factorization-based algorithms. The deformation energy kept by the first 15 bases (K=15) is 87.33%, which follows the imposed criteria of keeping 85% of the deformation energy.

#### 4.3.1. Performance Evaluation Based on Perfect Matching

**Figure 3:**
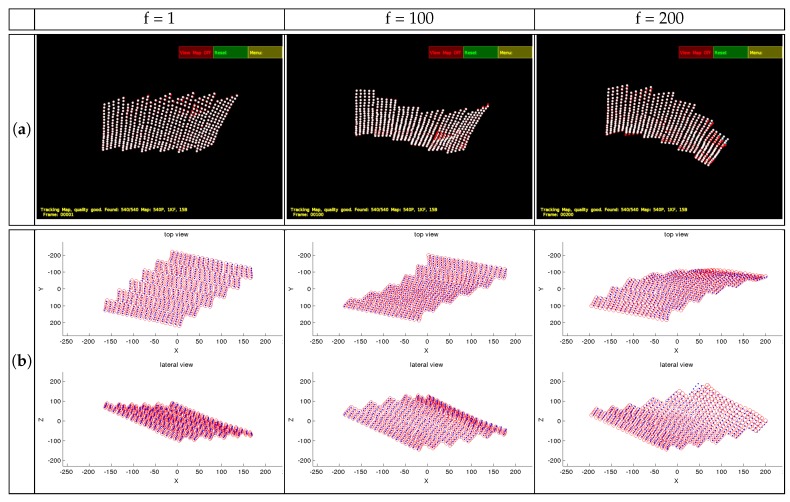
Flag sequence snapshots (**a**) and reconstruction results (**b**) for certain frames.

Some examples of both sequences are shown in Figure 3a. 3D reconstruction results without any degradation on the matching are shown in Figure 3b. They are compared to the ground truth, which is represented in blue, whilst the reconstruction result is represented in red. Two views are shown to better display the 3D reconstruction. For most of the frames, the reconstruction is very accurate. The average performance is shown in the row for K=15 bases shown in Table 1.

In Frame #200, the reconstruction result shows more error on one of the corners, which corresponds to high frequency deformations that might not have been properly modeled. The results can be improved, in this case of perfect matching, by increasing the number of bases.

**Figure 4:**
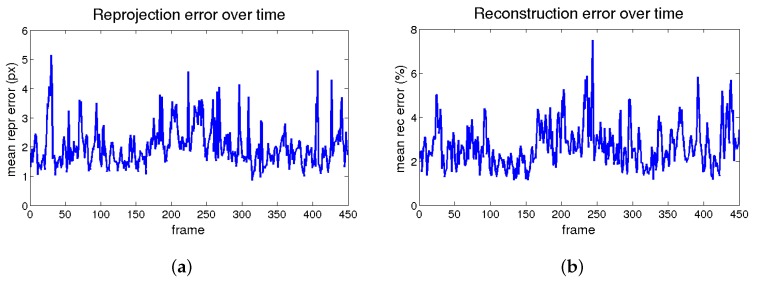
Flag sequence error figures through time. (**a**) The reprojection error and (**b**) the 3D reconstruction error.

The reprojection and reconstruction errors for a perfect matching over time are shown in Figure 4. Increasing the number of bases minimizes these errors, as will be seen in Section 4.3.4. However, as will be discussed in Section 4.6.2, this trend will not be valid when correspondences are obtained with the matching of real images.

#### 4.3.2. Performance Evaluation Based on Visibility Degradation

Missing detection of features is simulated by using visibility masks. These masks are generated according to a percentage of missing data. The visibility distribution of the masks for each point and among frames follows a uniform random distribution.

Some results within a range of visibility from 5%–100% are carried out. The 2D reprojection and the 3D reconstruction errors are computed for both sequences. For the flag sequence, the errors are stable from 20–100%, which represents a minimum of 108 points. They have the following values on average: two pixels for 2D reprojection error and 2.6% for 3D reconstruction error. We highlight that the visibility distribution follows here a random uniform distribution. In real images, missing data usually affect entire areas of the image, due to occlusions or lack of texture. In some detectors, the visibility percentage varies from 50%–30%.

#### 4.3.3. Performance Evaluation Based on Noise and Outliers

Similarly to the previous test, robustness analysis due to outliers and noise is performed. Different values for noise and outliers have been evaluated, as mentioned in Section 4.1. The experiments are repeated 10 times, and the average is taken, to achieve representative results, as a standard Monte Carlo experiment.

**Figure 5:**
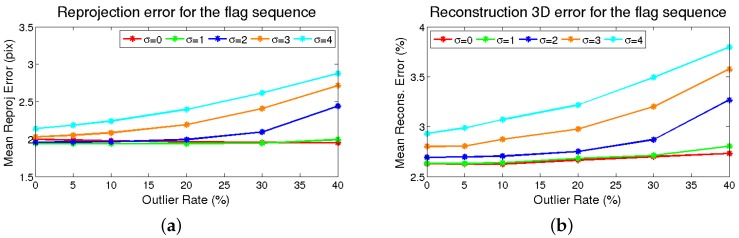
Flag sequence results for noise and outliers’ degradation on tracking. (**a**) The column depicts the error variation with the *o* outlier points’ percentage of the reprojection error. Each trace corresponds to a different noise σ level. (**b**) The column represents the same for the 3D reconstruction error.

The results are shown in Figure 5. It can be seen that the 2D reprojection error is not significantly increased for the lowest σ values as the number of the outliers grows, meaning that the M-estimator is capable of dealing with them.For higher values of σ, the error gap between the methods is approximately the same as the number of outliers increases, the limit being σ=2. With respect to the 3D reconstruction error, the trend is the same as in 2D reprojection error, starting at 2.6%.

#### 4.3.4. Performance Evaluation Based on the Number of Bases

The dependence of the accuracy results with the number of bases is checked as well. For that purpose, a test using tracks without degradation and with 5, 7, 15, 25 and 30 bases is presented in Table 1.

Using perfect matching, as the number of bases increases, the error decreases for the presented sequence and so do all the error indicators. In addition, the processing time improves as the number of bases is reduced, as well as the memory requirements.

#### 4.3.5. Comparison with Other Methods of the State-Of-The-Art

Table 2 shows a summary of results with the most representative methods of the state-of-the-art for the flag sequence. Processing time for the whole sequence, 3D accuracy, 2D error, rank, modeling type (auto/a priori) and procedure type (batch/sequential) are depicted. In this case, our algorithm gets close to the best ones in terms of 3D accuracy and 2D reprojection error for this sequence. In our approach, no priors are applied, in contrast to the best ones, in which physical priors such as isometry [34] or partial modeling [42] are used. Our approach can be better compared with sequential algorithms [21,24], as the estimation scheme follows a similar (sequential) approach. Regarding [21], our algorithm outperforms their 2D and 3D error. With respect to [24], our algorithm obtains comparable results using a lower amount of bases and without physical priors.

Regarding computation times, our algorithm is the best among all compared methods, thus reaching the best trade-off between performance and processing time. However, it must be highlighted that some of the competing methods are not optimized to run in real time, and some of them are implemented in MATLAB, while our method is implemented and optimized in C++.

The trials of [44,69] did not finish processing the sequence after the referenced time. For some of the methods, where the source code is available [11,12,20,21,44,69], the same hardware is used to run the experiments. In [69], they provide results with this dataset. We present them in a second column inside the 3D error column as we found some discrepancy betweenour tests and the ones provided in the original paper.

Algorithms where the code and processing times are not available, such as the results of [34,42], are not reported in Table 2. Batch algorithms implemented in MATLAB such as [11,12,20,21,24,44,69] take more time than those developed in the C++ language, like [40]. A C++ implementation does not automatically imply real-time performance, as the code needs to be optimized, but also the data volume needs to be restricted to be properly handled in real time.

Comparing the MATLAB processing time in Table 2 and Table 3, it gives an idea of the complexity of the flag dataset. Table 2 shows that current solutions based on model-free methods (NRSfM) cannot recover the correct 3D shape for the flag sequence with a non-normalized error, unless the approach includes some extra prior (we recommend to the readers the review of [69]). As our method is model-based, it provides acceptable error values and is faster than the rest of model-free approaches, reaching 70–90 fps, which is compatible with real-time constraints. It must be noted that no visual information processing is applied in any of the tested approaches.

### 4.4. CMUfaceSequence

This sequence presents a face of a person while changing the pose and talking. This sequence is used in other methods such as in [21]. The sequence is mostly subject to rigid motions, although it presents deformations on the mouth area, when the person is talking. The amount of points is 40 over 315 frames projected on an image size of 640 × 480. The deformation energy kept by the first 15 bases (K=15) is 95.8%, which follows the minimum energy criteria explained in Section 4.2.

The same tests have been carried out for this sequence, and the same trends observed in previous experiments have been seen here. As the number of points is significantly fewer than in the flag sequence, the minimum amount of points to consider for visibility is 50%. The same trend and conclusions can be extracted for the analysis with noise, outliers and bases, only varying the average values. The average value for K = 15 bases is 0.26 for 2D reprojection error and 1% of 3D reconstruction error.

Table 3 shows a summary of the results obtained with the CMUfacecompared to the sparse approaches [11,12,20,21]. The same format as in Table 2 is used. In this case, the presented algorithm gets the best results among the tested algorithms for the error parameters and the time performance.

In a sequence in which most of the behavior is rigid except for the mouth movements, the overall performance of the algorithms is good as they all exploit this circumstance. The presented model-based reconstruction algorithm outperforms the results of model-free approaches.

### 4.5. Point-Wise CVLab’s Kinect Paper

This dataset is derived from the Kinect paper dataset presented in [70]. It consists of a video sequence of 99 points on 191 frames showing a deforming piece of paper, which is captured with a depth sensor. The projections of the mesh for each frame have been obtained by interpolation from the set of original matches. The projections are further adjusted by estimating the relative camera-object pose and making sure that they match with the original SIFT images.

We see the same trend observed in the previous datasets when the number of bases is incremented. This dataset needs a bigger amount of bases to achieve good reconstruction results. For K = 15, the 2D error is about 9 px, and the 3D error almost reaches 11.92%, whereas, for K = 80, the 2D error becomes lower (8 px approximately), and the 3D error goes to 11.8%.

### 4.6. Rendered Flag Sequence

The flag sequence depicted in the previous tests is interpolated to get a dense surface and then is rendered using orthographic projection, as presented in [71]. As our model-based tracking works with the perspective projection, this sequence was re-rendered with perspective projection. The sequence contains 450 frames.

This sequence was chosen in other works [24,43,52,71] as it consists of a set of images and a dense 3D ground truth to evaluate the performance of tracking methods. It is useful for our experiments because, even though our tracking is sparse, the ground truth is appropriate to perform a thorough comparison among different methods.

#### 4.6.1. Evaluation of Visual Descriptors

**Figure 6:**
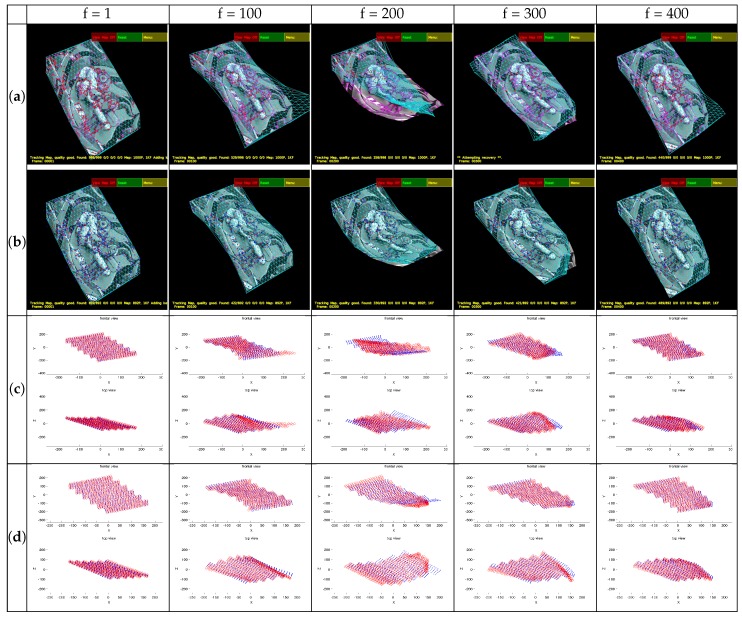
Rendered flag sequence screenshots and reconstructions for IROS and SIFT matching. (**a**) IROS screenshots, (**b**) SIFT screenshots, (**c**) IROS reconstruction results, (**d**) SIFT reconstruction result.

First of all, some frames of the sequence processed with the IROS-based tracking published by the authors in [52] are shown in Figure 6a together with reconstructions (Figure 6c). The same frames processed with the SIFT tracking and their reconstructions are shown in Figure 6b,d. The detected points (Figure 6a,b is shown in magenta, the matched points in red, and the mesh model is overlaid in cyan, to give an idea of the tracking performance. Regarding the 3D reconstructions (Figure 6c,d), the ground truth points are represented in blue and the reconstruction in red.

In our method, the feature detection and matching methods are configured to prioritize speed over accuracy. The frames are ordered in the columns and the tracking methods in the rows. It can be seen that in Frame #200, there are some accuracy problems for the presented approaches. This is due to the sequence presenting very abrupt movements combined with an interval of possible tracking losses. The detection of features in these areas is a difficult task, even for the most advanced feature descriptors, so there are very few points detected in this area, yielding a poor local estimation.

The projection of the model on the screenshots (cyan) and the reconstruction results differ because the shape in the latter is obtained after applying Procrustes alignment between the ground truth and the reconstruction.

In order to have a general performance overview for all the analyzed descriptors, a benchmark is shown in Table 4. This comparative includes number of points, descriptor, matcher, processing time (for the whole sequence and fps), 2D error and 3D error.

**Figure 7:**
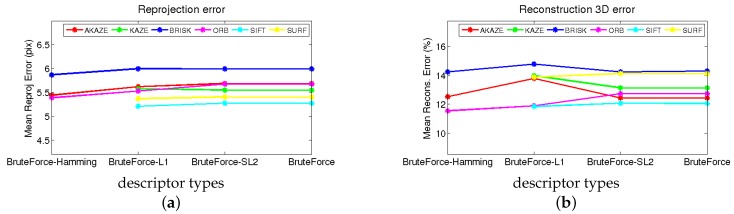
Rendered flag sequence rank based on the error results of the different descriptor methods. (**a**) Reprojection error; (**b**) 3D reconstruction error.

A similar benchmark, but including the matching technique used for each feature descriptor, is shown in Figure 7. This studies the influence of the matching method on the error metrics. For binary descriptors such as AKAZE, ORB and BRISK, the use of Hamming distance slightly improves the results of 2D reprojection and 3D reconstruction.

Figure 7 shows that the best method according to the reprojection error is not necessarily the best in terms of the reconstruction error, as we pointed out before. SIFT gets the best performance for both error estimations and among all the matching algorithms (brute force L1, L2). However, the second best is not the same for both figures (SURF in 2D and AKAZE in 3D).

AKAZE performs almost as well as SURF in terms of 2D reprojection error and is almost as good as SIFT in terms of 3D reconstruction error. Looking at Table 4, it can be seen that AKAZE is also one of the fastest approaches, which makes it a good candidate for a final implementation.

Regarding this table, it can be seen that the fastest approaches are IROS, AKAZE, ORB and SURF, and the ones that handle the most points are IROS, BRISK, ORB and SIFT. We remind that state-of-the-art approaches are only capable of working with a very small amount of points obtained from interpolated models. In this case, all the detected points are used to perform the estimation. It is important to remark that most of the reported time is consumed by the feature detection algorithm. The obtained times show the influence of the visual processing, compared with the reported results on the flag sequence in Table 2, in which the visual detection stage is not applied. PTAM results are included as a baseline for rigid SfM.

#### 4.6.2. Performance Evaluation Based on the Number of Bases

As was done previously with the point-wise flag sequence dataset, the influence of the number of bases is tested for real conditions.

We show averaged results in Table 5. It can be seen that the trend observed with ideal matching is reversed here. Increasing the number of bases does not necessarily increases the 3D error. For IROS-based and SIFT-based tracking, the best results are obtained for seven bases.

This clearly means that with real matches, the DoF of the shape model plays an important role in the reconstruction accuracy. With more bases, the model becomes more capable of adapting to large matching errors and more prone to erroneous reconstructions in areas of the object with a small amount of detected points. This effect is fixed by introducing priors as we explain in the next section and as displayed in Table 6.

#### 4.6.3. Performance Evaluation with Time and Shape Smoothing Priors

Working with real images increases uncertainty in matching, which causes ambiguities and errors in the 3D shape. In order to reduce these effects, priors must be added. Due to the nature of our optimization algorithm (linear and split), time and shape smoothness priors can be implemented. The priors are studied at three levels, depending on the strength at which they are imposed, which corresponds to the value of their hyperparameters in the optimization (low, medium and high). The values for each type of prior are obtained heuristically.

The SIFT descriptor is chosen for the matching, as it obtained the best performance results in our previous tests (Table 4). Then, the results are compared to the IROS approach because it serves as a baseline of our first implementation [52].

After 3D reconstructions and 2D projections have been studied, a temporal analysis of the tracking for both priors regarding the baseline without priors is carried out.

**Figure 8:**
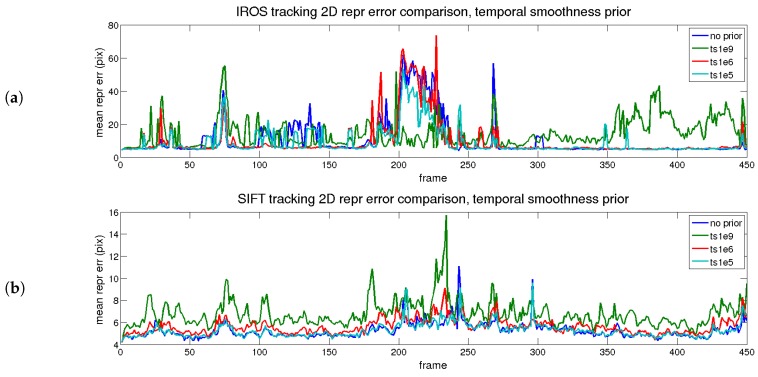
Rendered flag sequence: reprojection error results over time when the time smoothness prior is applied for both descriptor types. (**a**) IROS; (**b**) SIFT.

**Figure 9:**
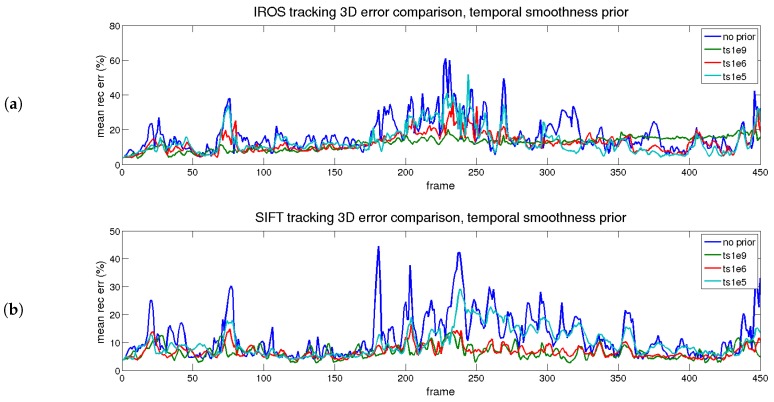
Rendered flag sequence: 3D reconstruction error over time with time smoothness. Results are shown for two types of descriptors. (**a**) IROS; (**b**) SIFT.

The first evaluated prior is time smoothness, depicting the errors shown in Figure 8 and Figure 9, which correspond to reprojection and 3D reconstruction errors. The figures are divided into two parts: the upper part corresponds to the IROS tracking, whereas the lower part corresponds to the SIFT tracking.

In Figure 8, the reprojection errors are presented for the different priors with different colors. The blue trace represents the result without priors. In terms of error, cyan (low) and red (medium) traces are close to the blue trace. The green trace (high) presents high error for both approaches, except when the tracking is lost, as an excessive value of the prior makes the shape too rigid. The cyan trace, as expected, has little effect on the reprojection error, as the regularization value is low. The red trace has a slightly higher reprojection error, although in general terms, it is maintained along the sequence. Therefore, for the reprojection error, the prior does not imply an improvement.

The beneficial effect of the prior is seen in terms of reconstruction error, shown in Figure 9. For both approaches, when the prior is applied (even for small values), the 3D reconstruction error is reduced.

The best option corresponds to the red trace (medium) for both approaches. In that case, the increment in the reprojection error is small and the improvement in the 3D reconstruction error is large with respect to the blue trace (no priors applied). The effect is even more noticeable when the tracking is better, comparing the IROS and SIFT approaches.

To sum up, the errors reached after applying different priors are depicted in Table 6. The best results for each prior are marked in bold.The best option is obtained for a combination of both priors with medium values.

**Figure 10:**
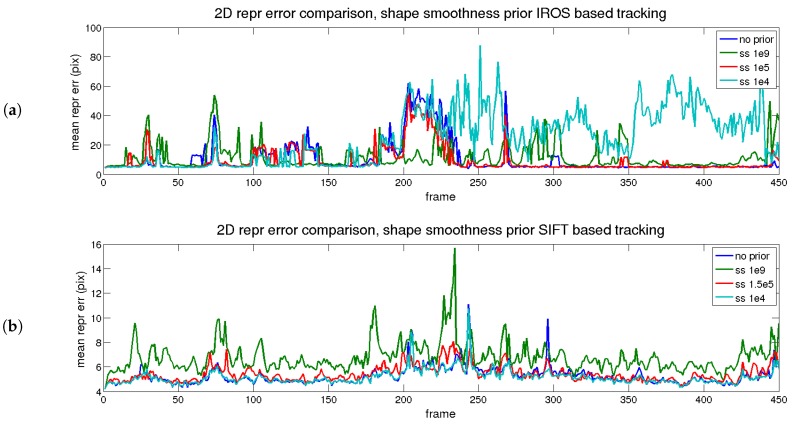
Rendered flag sequence: reprojection error over time with shape smoothness. Results are shown for two types of descriptors. (**a**) IROS; (**b**) SIFT.

**Figure 11:**
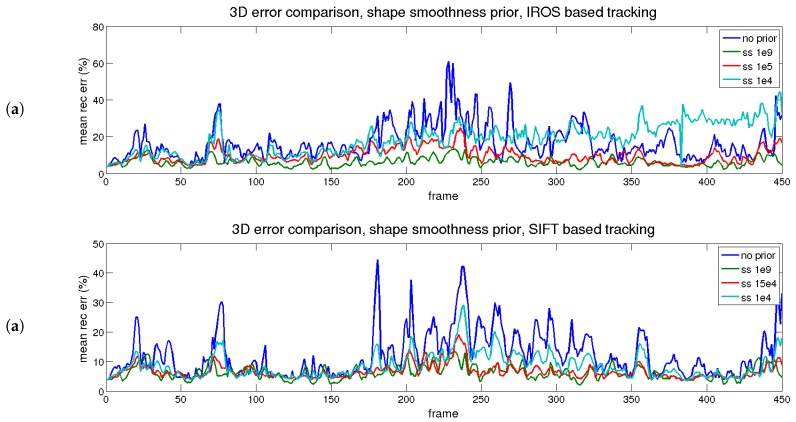
Rendered flag sequence: 3D reconstruction error over time with shape smoothness. Results are shown for two types of descriptors. (**a**) IROS; (**b**) SIFT.

The results of the temporal analysis for the shape smoothness prior are depicted in Figure 10 (reprojection) and Figure 11 (3D reconstruction). A similar conclusion to the temporal smoothness prior can be extracted in this case. The trace in red (medium) reflects an equilibrium between reprojection and reconstruction errors, for both matching methods (IROS- and SIFT-based).

PTAM results are included to have a rigid SfM baseline in this sequence. On the other hand, just on the row below, the results from the (point-wise) flag sequence with perfect matching (Table 2) are displayed to show the impact of dealing with real images.

According to Table 6, the difference between the IROS and SIFT approaches is easy to explain: tracking quality is decisive to get accurate results, as the 2D reprojection error is about 4.3 pixels lower for SIFT before priors’ application. Regarding the 3D reconstruction error, it is about 4% lower (16 down to 12) before priors, and after priors, it is reduced an additional 3% with respect to the best mark (9 down to 6).

Processing time due to the addition of priors is not included in the table, because it does not add a substantial change.

### 4.7. CVLab’s Kinect Paper

This sequence was first proposed in [70]. It consists of 191 frames of a well-textured piece of paper undergoing deformations and pose changes. The dataset includes a dense ground truth for each frame in the sequence. These data are acquired using the Kinect sensor, commonly used to test SfT algorithms.

One of the main differences of this sequence compared to the rendered flag is that, in this case, the background contains texture. Therefore, the area of the object is segmented at the beginning of the sequence to reject background features.

Regarding the results, they are similar to the ones in the rendered flag sequence. As the number of bases is increased, the 2D error is reduced, and the 3D error is increased. This time, there are few differences between the results using IROS and SIFT matching, as we show in Table 7.

The results are this time slightly better for the IROS approach compared to SIFT as the search radius is greater in the latter. The possibility of having incorrect matches due to a bigger radius is increased even though SIFT matching is better.

Finally, a comparative table with other state-of-the-art algorithms is shown in Table 8. We show our results without smoothing priors, which is a conservative result (check Table 7). It can be seen that our results are comparable with those obtained in [72].

## 5. Conclusions

This paper has presented a real-time and model-based reconstruction algorithm for non-rigid objects that offers a good trade-off between speed and quality. Our algorithm is based on PTAM, a well-known state-of-the-art SfM method that works for rigid objects. We upgrade PTAM’s tracking thread to solve SfT using a low-rank shape basis model. We keep PTAM’s real-time performance.

We demonstrate that our tracking algorithm is comparable in terms of accuracy to some of the most representative non-rigid reconstruction methods. It deals with challenging imaging conditions such as visibility reduction, outliers and noise. These conditions are not tackled by the majority of the state-of-the-art methods as they work with pre-registered image data. Our outlier rejection algorithm, based on [8], is effective for datasets showing mostly rigid or isometric deformations. Further tests on elastic sequences are thus required.

Our method includes different feature matching methods that improve tracking quality in real images. SIFT has been demonstrated to be the best matching method in terms of 2D reprojection and 3D reconstruction error. However, it is very slow to comply with real-time constraints. The best trade-off between accuracy and complexity is given by the AKAZE matching method.

Our method uses priors for spatial and temporal smoothness. We show that a combination of the two priors helps to reduce the flickering, which is noticeable on the 3D reconstruction error and stabilizes the estimated trajectory.

Our current implementation requires further code optimizations to reduce CPU load (redundancy checks and code profiling). The objective is to reach stable 30 or even 60 fps for VGA images with shapes consisting of thousands of points.

Future research might include dense matching and shape representation. Our tracking method is also a key component for proposing a sequential NRSfM algorithm.

## Figures and Tables

**Table 1:** Flag sequence error comparison with the number of bases, for K = 5, 7, 15, 25 and 30.

#Bases (*K*)	2D Error (pix)	3D Error (%)
5	4.56	3.84
7	3.62	3.21
15	2	2.63
25	1.34	2.1
30	1.18	1.93

**Table 2:** Flag sequence results summary. Some results comparing several state-of-the-art algorithms and the presented one are depicted.

Method	2D Error (px)	3D Error (%)	Rank	Tproc (fps)	Model	ProcType	Impl.
[21]	6.14	91.65	7 (max)	20 min (0.37)	auto	seq.	MATLAB
[20]	29.79	66.65/16.09 ^1^	5	36 min (0.21)	auto	batch	MATLAB
[11]	11.65	15.59/13.25 ^1^	4	10 h (0.01)	auto	batch	MATLAB
[12]	**1.9**	29.724/17.61 ^1^	9	19 seg(23.68)	auto	batch	MATLAB
[34]	-	**1.79**	-	-	auto	batch	MATLAB
[42]	-	**1.29**/3.25 ^1^	-	-	auto	batch	MATLAB
[69]	**~2**	n.a./3.8	-	>>28 h	auto	batch	MATLAB
[44]	-	n.a./17.41 ^1^	-	>>16 h	auto	batch	MATLAB
[24]	-	3.28 (K = 10) 2.81 (K = 40)	10–40	708 seg (K = 10) (0.64) 1045.5 seg (K = 40) (0.43)	auto	seq.	MATLAB
Our approach	**2**	**2.63**	15	**5 seg. (90)**	priory	seq.	C++

^1^ In [69], the authors report other values of 3D reconstruction methods [11,12,20,42,44]. They claim they tuned the setup to be optimal, but they neither specify how long the experiments took, nor the setup of their trials, nor the configuration parameters.

**Table 3:** CMUfacesequence results summary. Some results comparing different state-of-the-art methods against the presented one are depicted.

Method	2D Error (px)	3D Error (%)	Rank	Tproc (fps)	Model	Proc Type	Impl.
[21]	1.06	3.18	8 (max)	14 min (0.37)	auto	seq.	MATLAB
[20]	0.6	3.19	5	43 seg. (7.35)	auto	batch	MATLAB
[11]	6.42/32.39	9.9/56.06	5/15	78/606 seg. (4.05/0.52)	auto	batch	MATLAB
[12]	1.06	2.43	12	13 seg (24.3)	auto	batch	MATLAB
Our approach	**0.26**	**1.01**	15	**4.5 seg. (69.78)**	priory	seq.	C++

**Table 4:** Rendered flag sequence descriptor results comparison. As references, the original PTAM algorithm, the PTAM-based tracking and the descriptor-based results are presented.

Descriptor	Matcher	2D Error (px)	3D Error (%)	tp (s/fps)	Map Pts
PTAM	PTAM	40.12	104.54	102/4.4	350
IROS	PTAM-like	9.51	16.65	**26/17.3**	972
AKAZE	Brute force	5.68	12.42	**27/16.6**	607
BRISK	Brute force	5.98	14.27	56/8	1000
ORB	Brute force	5.67	12.71	29/15.5	1000
KAZE	Brute force	5.55	13.12	49/9.2	347
SIFT	Brute force	**5.27**	**12.04**	87/5.2	892
SURF	Brute force	5.4	14.11	29/15.5	650

**Table 5:** Rendered flag sequence: performance vs. the number of bases for IROS- and SIFT-based tracking.

Matching	#Bases	2D Error (pix)	3D Error (%)
**IROS**	**7**	**8.83**	**9.19**
IROS	15	9.51	16.65
IROS	30	13.2	34.23
SIFT	7	5.76	**6.53**
SIFT	15	5.27	12.04
SIFT	30	**5.23**	17.91

**Table 6:** Summary of the results for the rendered flag sequence with different numbers of bases, priors and descriptors.

Desc.	Prior Type	Value	2D Error (px)	3D Error (%)
PTAM	none	0	40.12	104.54
Point-wise ideal Matching	none	0	2	2.63
IROS	none	0	9.51	16.65
IROS	time	1e5	8.92	13.38
**IROS**	**time**	**1e6**	**9.76**	**12.42**
IROS	time	1e9	14.37	12.04
IROS	shape	1e4	24.05	18.31
**IROS**	**shape**	**1e5**	**9.38**	**9.54**
IROS	shape	1e9	11.62	6.2
**IROS**	**both**	**1e5/1e5**	**8.72**	**8.95**
SIFT	none	0	5.27	12.04
SIFT	time	1e5	5.29	9.93
**SIFT**	**time**	**1e6**	**5.64**	**6.79**
SIFT	time	1e9	6.79	6.51
SIFT	shape	1e4	5.18	8.48
**SIFT**	**shape**	**1.5e5**	**5.45**	**6.56**
SIFT	shape	1e9	6.80	6.2
SIFT	**both**	**1e5/1.5e5**	**5.48**	**6.46**

**Table 7:** Summary of the results for the Kinect paper sequence with different numbers of bases, priors and descriptors.

Desc.	Prior Type	Value	#Bases	2D Error (px)	Depth Error (mm)
Point-wise ideal Matching	none	0	15	9.06	10.39
Point-wise ideal Matching	none	0	50	7.98	10.35
IROS	none	0	15	26.28	10.34
IROS	time	1e3	15	26.45	14.34
IROS	shape	1e4	15	27.48	10.38
IROS	none	0	50	24.77	11.44
IROS	time	1e3	50	24.82	11.37
IROS	shape	1e4	50	24.35	10.68
SIFT	none	0	15	23.34	10.63
SIFT	time	1e3	15	23.36	10.60
SIFT	shape	1e4	15	23.43	10.48
SIFT	none	0	50	24.47	13.64
SIFT	time	1e3	50	24.45	13.67
SIFT	shape	1e4	50	23.62	11.17

**Table 8:** Comparative results with other state-of-the-art algorithms in the Kinect paper sequence.

Method	Depth Error (mm)	Rank	Tproc (fps)	Model	Proc Type
[72]	7.23	-	-	priory	batch
[72] (PCA)	11.68	-	-	priory	batch
[73]	6.9	-	-	priory	sequential
[74]	5.57	-	959 (0.2)	priory	sequential
Our approach (SIFT)	13.65	50	90 (2)	priory	sequential
Our approach (IROS)	11.44	50	17 (11.24)	priory	sequential
